# Saliva as an alternative specimen to nasopharyngeal swabs for COVID-19 diagnosis: Review

**DOI:** 10.1099/acmi.0.000366

**Published:** 2022-05-20

**Authors:** Leah McPhillips, John MacSharry

**Affiliations:** ^1^​ School of Microbiology, University College Cork, Cork, Ireland; ^2^​ School of Medicine, University College Cork, Cork, Ireland; ^3^​ The APC Microbiome Ireland, University College Cork, Cork, Ireland; ^†^​ Present address: Department of Molecular Microbiology, The John Innes Centre, Norwich, UK

**Keywords:** Saliva, COVID-19, diagnosis, SARS-CoV-2, Nasopharyngeal swabs

## Abstract

Almost 2 years ago, the novel coronavirus, severe acute respiratory syndrome coronavirus 2 (SARS-CoV-2) was discovered to be the causative agent of the disease COVID-19. Subsequently, SARS-CoV-2 has spread across the world infecting millions of people, resulting in the ongoing COVID-19 pandemic. The current ‘gold standard’ for COVID-19 diagnosis involves obtaining a nasopharyngeal swab (NPS) from the patient and testing for the presence of SARS-CoV-2 RNA in the specimen using real-time reverse transcription PCR (RT-qPCR). However, obtaining a NPS specimen is an uncomfortable and invasive procedure for the patient and is limited in its applicability to mass testing. Interest in saliva as an alternative diagnostic specimen is of increasing global research interest due to its malleability to mass testing, greater patient acceptability and overall ease of specimen collection. However, the current literature surrounding the sensitivity of saliva compared to NPS is conflicting. The aim of this review was to analyse the recent literature to assess the viability of saliva in COVID-19 diagnosis. We hypothesize that the discrepancies in the current literature are likely due to the variations in the saliva collection and processing protocols used between studies. The universal adaptation of an optimised protocol could alleviate these discrepancies and see saliva specimens be as sensitive, if not more, than NPS for COVID-19 diagnosis. Whilst saliva specimens are more complimentary to mass-testing, with the possibility of samples being collected from home, the RT-qPCR diagnostic process remains to be the rate-limiting step and therefore interest in salivary rapid antigen tests, which negate the wait-times of RT-qPCR with results available within 15–30 min, may be an answer to this.

## Introduction

The on-going COVID-19 pandemic has drastically impacted all corners of the globe. The rise of new and more infectious severe acute respiratory syndrome coronavirus 2 (SARS-CoV-2) variants and with the majority of the world population still to receive vaccination against the virus, testing, social-distancing, face-coverings, self-isolation/quarantining and regional/country-wide lockdowns remain to be the modus operandi in controlling viral spread [1, 2]. For COVID-19 diagnosis, the World Health Organization (WHO) currently recommends real-time reverse transcription PCR (RT-qPCR) to detect SARS-CoV-2 RNA from nasopharyngeal and/or oropharyngeal swabs (NPS and OPS), with NPS being the reference standard for routine diagnosis [3]. This is primarily because NPS have been established as the specimen of choice for the diagnosis of other viral respiratory infections [4, 5]. However, NPS have a number of limitations including the requirement of trained health-care workers (HCWs), personal protection equipment (PPE), swabs and viral transport media (VTM) for specimen collection which has resulted in global supply shortages and testing ‘bottlenecks’ [6–8]. NPS collection is an invasive and uncomfortable procedure which can lead to physical resistance thus affecting specimen quality, cause bleeding in patients with thrombocytopenia and ultimately makes individuals less likely to return for future/repeated testing [9–12]. This can also impact NPS’s applicability to COVID-19 surveillance, as repeated swabbing can leave patients with symptoms such as nosebleeds, headaches, rhinorrhoea and general nasal discomfort that can last up to 24 h [13]. Additionally, NPS collection puts HCWs at high risk of nosocomial infection as specimen collection can induce coughing/sneezing in patients [14]. Therefore, alternatives to NPS have become of global research interest, where in particular saliva has come to the fore. Saliva offers a number of advantages over the current reference standard including the less invasive nature of specimen collection creates greater patient acceptability and thus individuals are more likely to get tested and give repeated specimens [15]. It does not require close-contact with HCWs thus reducing the risk of nosocomial infection and demands for PPE. Saliva collection also has the potential to be self-administered from home and is not affected by supply shortages as only a sterile container is required for specimen collection [16]. Currently, the WHO does not recommend saliva as the only specimen for routine diagnosis, but it is used for COVID-19 surveillance in Hong Kong and parts of the USA where a number of saliva-based assays have been granted Emergency Use Authorisation (EUA) by the US Food and Drug Administration (FDA), where it has in particular been adopted to carry out surveillance in educational institutes [17–24]. In this review the methods of saliva specimen collection, transport and processing, its accuracy compared to NPS especially for mild/asymptomatic patients, its potential to be implemented for mass screening/surveillance and the limitations both of saliva as a diagnostic tool and the corresponding literature will be discussed.

## Main

### Saliva specimen collection, transportation and storage and their impact on specimen sensitivity

There are a number of possible sources of SARS-CoV-2 in saliva [25]. It was initially uncertain whether SARS-CoV-2 directly infected epithelial cells in the oral cavity although the SARS-CoV-2 receptor, angiotensin-converting enzyme 2 (ACE2), and both the transmembrane serine protease (TMPRSS) receptors, TMPRSS2 and TMPRSS4, which are required for spike protein cleavage to allow receptor binding and membrane-fusion to facilitate viral entry into the host cell, are highly expressed on the surface of the minor salivary gland epithelial cells and the oral mucosa [26–32].Whilst furthermore, SARS-CoV, the causative agent of the SARS outbreak in 2002–2004, was found to infect salivary gland cells in a Rhesus macaque model [33]. It has recently been confirmed through both COVID-19 patient autopsies and mildly symptomatic COVID-19 outpatient samples that SARS-CoV-2 can directly infect the epithelial cells of the salivary glands and oral mucosa [34]. This not only explains symptoms such as loss of taste, dry mouth etc. experienced by many COVID-19 patients but also further strengthens the argument for using saliva as a COVID-19 diagnostic by providing a definitive source of the virus in saliva. Other potential sources of SARS-CoV-2 in saliva include entry by the exchange of fluids, liquid droplets and/or the mucociliary escalator from the nasal cavity and/or the respiratory tract [35]. SARS-CoV-2 present in the blood could also enter saliva via gingival crevicular fluid and indeed has been detected in gingival crevicular fluid, or by salivary epithelial cells transporting blood-borne virions across the epithelial barrier by endocytosis and exocytosis [36, 37].

**Fig. 1.**
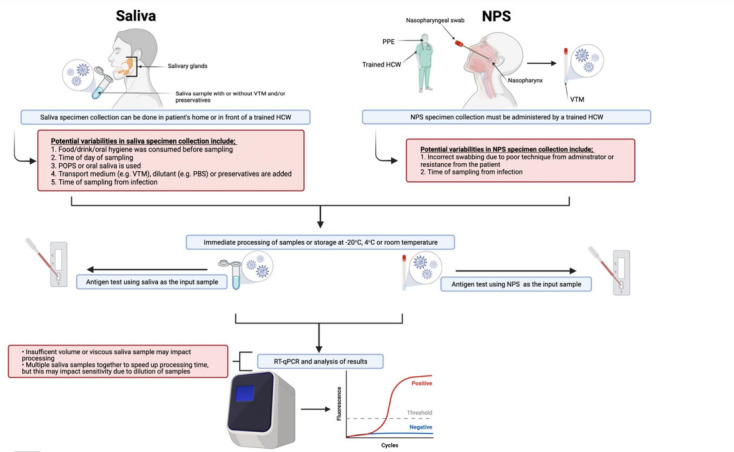
Overview of saliva and NPS specimen collection and processing for COVID-19 diagnosis including potential pitfalls in sample collection and processing (red) that could lead to variability in the specimen’s sensitivity.

**Fig. 2.**
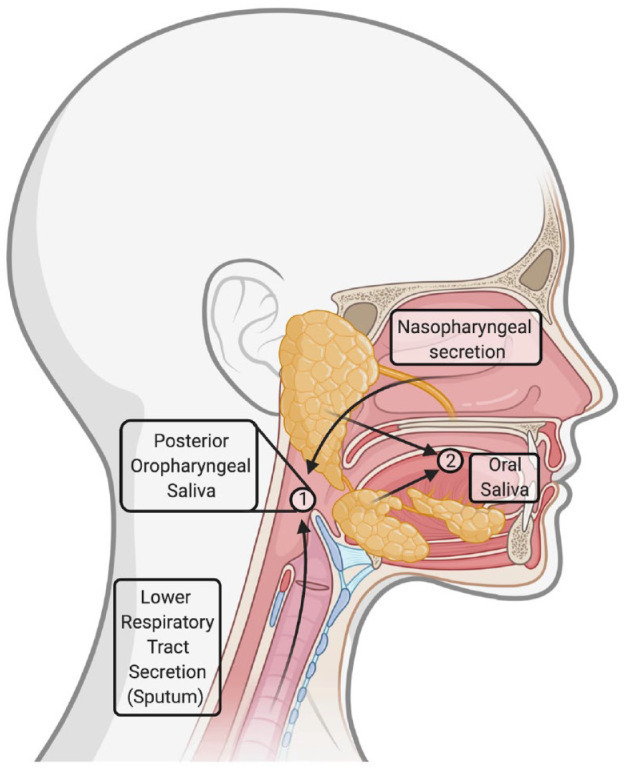
Source and location of posterior oropharyngeal saliva and oral cavity saliva. Taken from [105] with permission.

A large number of studies have been conducted since the COVID-19 pandemic began to determine the efficacy of saliva for COVID-19 diagnosis against the reference standard NPS (Table 1). The results from these studies have varied with some finding saliva to be equal to or more sensitive than NPS [38–48] whereas others found it to be less sensitive [49–61]. The specimen collection protocol is one factor that could result in the inconsistencies between these studies (Fig. 1). The method of saliva collection in these studies has varied from posterior oropharyngeal saliva (POPS), or deep throat saliva, obtained by the patient clearing their throat [15, 19, 35, 39, 40, 42, 43, 56, 62, 63] (Fig. 2), to saliva from the oral cavity obtained by spitting/drooling [38, 47, 51, 52, 55, 59, 64–71] (Fig. 2), swabs of the saliva glands [45, 57, 72, 73] and others not specifying the collection method [41, 49, 50, 53, 54, 58]. Currently, it is unclear whether POPS or oral saliva is the more sensitive specimen, although POPS likely has a higher viral load, particularly in the early morning, as it has possibly mixed with nasopharyngeal and bronchopulmonary secretions which move towards the posterior oropharyngeal region when individuals are lying down during sleep [35] (Fig. 2). However, POPS requires coughing/throat-clearing for specimen collection and therefore results in aerosolization of potentially contaminated aerosols and dry coughs are reported in around 80 % of COVID-19 patients which could result in difficulty in specimen collection [66, 74]. Additionally, POPS is more likely to be more mucus-rich and viscous which can create difficulties in saliva specimen processing (discussed further below), which is likely why [15, 35, 40, 56] added VTM to their POPS specimens. To the best of our knowledge, no study has directly compared the sensitivity between different saliva collection methods and therefore future research is needed to identify the best collection method for optimum saliva sensitivity [75].

**Table 1.** Studies that have evaluated the specificity of saliva specimens to NPS/other respiratory specimens. Adapted from [131] and [105]

Study and reference	Date	No. of paired samples	Patient information	Respiratory sample	SARS-CoV-2 target genes	Saliva sample (RT-qPCR) Sensitivity (%)	Discordance	Saliva specimen collection method	Ct value higher in saliva than respiratory sample
To *et al*. [15]	Feb-20	12	Confirmed hospitalised COVID-19 patients	NPS or sputum	S gene	11/12, 91.7%	one NPS/sputum only	Self-collected POPS, 2 ml of VTM added	Not mentioned
Chen *et al.* [57]	Mar-20	31	Confirmed COVID-19 patients – four had severe COVID-19	OPS	N gene and ORF1ab	4/31, 12.9%	27 OPS only	Saliva was collected from the opening of the salivary glands	Not mentioned
Wyllie *et al.* [38]	Apr-20	29 33	Severe hospitalised COVID-19 patients Asymptomatic at risk HCWs	NPS	N gene (N1 and N2)	Not mentioned, just stated saliva was more sensitive	two of the HCWs, two samples were saliva positive and all were NPS negative	Early morning before eating/brushing - self-collected by repeated spitting. Stored at room temperature.	No
Williams *et al.* [52]	Apr-20	522	Unconfirmed ambulatory outpatients	NPS	Not mentioned	33/39, 84.62%	one saliva only, six NPS only	Asked to pool saliva in mouth for 1–2 mins and then spit. A 1 : 1 ratio of Amies medium was added after.	Yes
Kojima *et al.* [132]	Apr-20	45	Unconfirmed patients - 21 were symptomatic	NPS	N gene (N1 and N2)	26/29 89.7%	Not mentioned	Clinician-supervised oral fluid swab specimens. Asked to enhance specimen by coughing before collection.	Yes
Becker *et al.* [58]	May-20	88 24	symptomatic convalescent	NPS	ORF1ab, S and N genes	69.2% 20–50 %	Not mentioned	Not mentioned but added stabilising solution	Not mentioned
McCormick-Baw *et al.* [64]	May-20	156	Hospitalised confirmed COVID-19 patients	NPS	N2 and E gene	47/49, 95.92%	one saliva only, three NPS only	Not mentioned but no preservative added	Yes
To *et al.* [35]	May-20	23	hospitalised confirmed COVID-19–10 had severe and 13 had mild symptoms	NPS or sputum	RdRP gene	20/23, 86.9%	three NPS/sputum only	POPS - early morning before eating/brushing - VTM added	Not mentioned
Pasomsub *et al.* [66]	May-20	200	Symptomatic unconfirmed patients	NPS and OPS	N gene and ORF1ab	16/19, 84.21%	two saliva only, one NPS only	Oral cavity saliva sample, collected without inducing coughing/throat clearing	Yes
Iwasaki *et al.* [68]	Jun-20	76	Mild-moderate symptoms of which 10 were confirmed COVID-19 patients	NPS	N gene	9/10, 90%	one saliva only, one NPS only	Self-collected oral saliva	Yes
Jamal *et al.* [55]	Jun-20	52	Confirmed hospitalised COVID-19 patients	NPS	RdRP, E and N gene	31/47, 77%	five saliva only, 11 NPS only	Asked to spit one teaspoon of saliva - 2.5 ml of PBS added, frozen at −80 °C	Mean difference not statistically significant
Chen *et al.* [56]	Jun-20	58	Confirmed COVID-19 patients	NPS	N2 and E genes	52/58, 89.7%	three saliva only, six NPS only	POPS - early morning before eating/brushing - 2 ml VTM added	Yes
Wong *et al.* [19]	Jun-20	229	229 paired NPS-saliva samples from 95 patients. 51 were COVID-19 confirmed of which seven were asymptomatic.	NPS	E gene	141/159, 88.7%	37 saliva only, 18 NPS only	POPS- early morning before eating/brushing – 1 ml of VTM added	Not mentioned
Chau *et al.* [53]	Jun-20	30	Confirmed COVID-19 patients −13 were asymptomatic and 17 were symptomatic	NPS	E gene	20/27, 74%	one saliva only, seven NPS only	Not mentioned	Yes
Griesmer *et al.* [54]	Jun-20	463	236 unconfirmed asymptomatic and symptomatic patients 227 unconfirmed symptomatic patients	NPS	N gene (N1)	6/12, 50 % 81/93, 87.1%	1) six NPS only 2) two saliva only, 12 NPS only	Not mentioned. Asked not to eat/drink/smoke 30 mins before saliva collection. Stored at 4 °C before processing	Yes
Skolimowska *et al.* [67]	Jun-20	132	Unconfirmed symptomatic HCWs and their household contacts	Combined NPS and OPS	ORF1ab and ORF8	15/18, 83.3%	one saliva only, three NPS only	Self-collected, patients asked to spit without inducing coughing	Yes
Azzi *et al.* [69]	Jul-20	25	Confirmed severe COVID-19 patients	NPS	5’ UTR	25/25, 100%	n/a	Oral saliva collected by drooling technique - or collected by pipette if patient was intubated	Not mentioned
Leung *et al.* [40]	Jul-20	95	95 paired saliva-NPS samples from 62 patients of which 29 were confirmed COVID-19 and 33 were COVID-19 negative patients	NPS	E gene	51/61, 83.6 %	13 saliva only, seven NPS only	Deep throat saliva (POPS) - 2 ml of VTM added	Not mentioned
Nagura-Ikeda *et al.* [82]	Jul-20	103	Confirmed COVID-19 of which 15 asymptomatic and 88 symptomatic	NPS or OPS	N1 and N2 genes	84/103, 81.6%	Not mentioned	Self-collected oral saliva, PBS added - no restriction on time of collection or of eating before collection	Not mentioned
Byrne *et al.* [85]	Jul-20	145	Unconfirmed symptomatic outpatients	Nasal/throat swabs	Not mentioned	20/23, 86.9%	three saliva only, three nasal/throat only	Pooled saliva and spat into sterile container – approximately 200 µL were collected and stored at −80 °C	Not mentioned
Landry *et al.* [70]	Jul-20	124	Unconfirmed symptomatic outpatients	NPS	N gene (N1 and N2)	30/35, 85.7%	two saliva only, five NPS only	Pooled saliva in mouth then spat into sterile container. Asked not to eat/drink 30 min before collection	Yes
Caulley *et al.* [50]	Aug-20	1939	High risk asymptomatic and mildly symptomatic unconfirmed patients	NPS or OPS	E gene	48/70, 68.6%	14 saliva only, 22 NPS only	Not mentioned	Not mentioned
Kim *et al.* [14]	Aug-20	15	Confirmed hospitalised COVID-19 patients, both asymptomatic and symptomatic	NPS or OPS	Not mentioned	34/53, 64 % (multiple samples taken)	Not mentioned	Oral cavity saliva - patients asked to spit into sterile container	Mean difference not statistically significant
Migueres *et al.* [51]	Aug-20	123	Confirmed COVID-19 patients both asymptomatic and symptomatic. 9 were hospitalised	NPS	RdRP gene	34/41. 82.93%	three saliva only, seven NPS only	Patients asked to swirl saliva in mouth for at least 30 s and then spit into sterile container	Yes
Rao *et al.* [39]	Aug-20	217	Confirmed asymptomatic male COVID-19 patients	NPS	E and RdRP genes	149/146, 93.1%	76 saliva only, 11 NPS only	Deep throat saliva (POPS) collected early morning before eating/brushing. Stored at room temperature before processing	No
Bhattacharya *et al.* [99]	Sep-20	74	Unconfirmed COVID-19 patients with mild to moderate symptoms	NPS	ORF1 and E genes	48/53, 90.5%	five NPS only	Not mentioned	No
Yokota *et al.* [41]	Sep-20	161 1763	1)Asymptomatic close-contact cohort 2) Asymptomatic airport quarantine cohort	NPS	N gene (N2 primer)	44/47, 93.6 % 4/5, 80 %. Total: 48/52, 92%	six saliva only, three NPS only one NPS only	Not mentioned but stored at 4 °C	Mean difference not statistically significant
Moreno-Contreras *et al.* [133]	Sep-20	253	Unconfirmed symptomatic outpatients 182 had a saliva and OPS sample taken 71 had saliva and both NPS and OPS	NPS and/or OPS	E gene	69/80, 86.2 % 25/34, 73.5%	28 saliva only, 11 OPS only six saliva only, nine NPS/OPS only	Self-collected, patients asked to spit repeatedly until 2–3 ml was collected. No VTM or stabilisers added	No
Nacher *et al.* [49]	Sep-20	776	Unconfirmed - 39 % were asymptomatic and 61 % were mildly symptomatic	NPS	RdRP, E and N genes	86/162, 53%	ten saliva only, 76 NPS only	Not mentioned but pooled saliva samples from multiple patients for testing	Not mentioned
Zhu *et al.* [98]	Sep-20	944	12 separate cohorts	NPS	Not mentioned	397/442, 89.82%	15 saliva only, 60 NPS only	Not mentioned	Not mentioned
Fan *et al.* [62]	Oct-20	65	Confirmed COVID-19 patients, of which 42 had severe COVID-19	Throat/nasal swabs	ORF1ab	37/42, 88.09%	Not mentioned	POPS. Patients asked not to eat/drink at least 30 mins before specimen collection	No
Hanson *et al.* [123]	Oct-20	354	Unconfirmed symptomatic patients	NPS	RdRP gene	81/86, 84.2%	six saliva only, five NPS only	Pooled saliva in mouth and spit repeatedly until approximately 1 ml was collected – asked not to induce coughing	Not mentioned
Berenger *et al.* [59]	Oct-20	75	Confirmed COVID-19 patients, of which 9.5 % were hospitalised	NPS or OPS	E and RdRP genes	58/75, 77.3%	six saliva only, 11 NPS only	Pooled saliva in mouth for 1–2 mins and then spit, and 3 ml of UTM was added	Yes
Senok *et al.* [71]	Oct-20	401	Unconfirmed symptomatic and asymptomatic patients	NPS	RdRP and N genes	28/35, 80%	nine saliva only, seven NPS only	Asked to pool saliva in mouth for 1–2 min and then spit saliva into sterile container. No transport media was added and patients were asked not to eat/drink/smoke at least an hour before hand.	Mean difference not statistically significant
Vaz *et al.* [84]	Oct-20	149	Symptomatic HCWs and confirmed COVID-19 patients	NPS	RdRP and E genes	69/73, 94.5%	two saliva only, four NPS only	Asked to spit 2 ml of saliva into sterile container. Saliva was diluted with PBS and transported at 4^ °^C and stored at −80 °C	Not mentioned
Procop *et al.* [42]	Nov-20	216	Unconfirmed symptomatic patients	NPS	N gene (N1, N2 and N3 primers)	100%, 38/38	n/a	POPS - Enhanced saliva sample by strong sniff and elicited cough - no transport media added	Yes
Kandel *et al.* [83]	Nov-20	432	Unconfirmed outpatients - 30 % asymptomatic	NPS	E gene	42/46, 91%	three saliva only, four NPS only	Pooled saliva in mouth for 60 secs. Asked to collected as much saliva as they could, up to 5 ml - no VTM added, stored at 4 °C until processing.	Yes
Yee *et al.* [65]	Nov-20	300	Unconfirmed symptomatic and asymptomatic and 27 were known COVID-19 patients	NPS	N, S and ORF1ab genes	79/97, 81.4%	ten saliva only, 18 NPS only	Asked to rub cheeks to stimulate saliva and then spit without coughing - asked to avoid eating/drinking/smoking at least 30 mins before collection	Yes
Matic *et al.* [86]	Nov-20	74	Symptomatic unconfirmed patients - included patients from long-term care facilities, HCWs and household close-contacts	NPS	E gene	15/21, 71.4%	one saliva only, six NPS only	Asked to pool saliva in their mouth and spit approximately 1 ml into a sterile container - stored at room temperature with no VTM added	Not mentioned
Braz-Silva *et al.* [45]	Dec-20	201	Unconfirmed symptomatic patients	NOP (nasal and oropharyngeal swab combined)	E and S genes	55/70, 78.6%	18 saliva only, 15 NOP only	Saliva was collected using the Salivette️ cotton pad device where patients were asked to chew down on cotton pad carefully for 1 min. Samples were collected in the morning before eating and were stored at 4^ °^C until processing.	No
Trobajo-Sanmartín *et al.* [60]	Jan-21	636	Unconfirmed symptomatic and asymptomatic patients	NPS	E gene	171/327, 51.9%	three saliva only, 156 NPS only	Asked to pool saliva in their mouth first - asked to avoid eating/drinking at least 1 h before collection	Yes
Ediz Tutuncu *et al.* [44]	Jan-21	53	Asymptomatic and mildly symptomatic confirmed COVID-19 patients	NPS	RdRP gene	48/53. 90.57%	five NPS only	Asked to spit between 3–4 ml of saliva – PBS was added	Mean difference not statistically significant
Goldfarb *et al.* [134]	Jan-21		Confirmed COVID-19 patients	NPS	RdRP and E genes	26/33, 79%	one saliva only, six NPS only	Asked to pool saliva and spit repeatedly a minimum of 5–10 ml	Yes
Hamilton *et al.* [76]	Jan-21	128	Unconfirmed asymptomatic patients	Undiluted NPS Diluted NPS	ORF1ab, N and S genes	11/19, 57.89%	eight NPS only three saliva only, five NPS only	Used OMNIgene sample collection tubes and buffer - asked to avoid eating/drinking/smoking at least 30 mins before collection	Yes
Babady *et al.* [63]	Jan-21	87 100	Unconfirmed symptomatic or close-contact patients	NPS OPS	N gene (N1 and N2)	16/17, 94.12% 29/35, 82.86%	one saliva only, one NPS six saliva only, one OPS only	POPS. Stored at room temperature before processing	Mean difference not statistically significant
Echavarria *et al.* [135]	Feb-21	174	Unconfirmed symptomatic patients	NPS	E gene	61/63, 98%	one saliva only, two NPS only	Self-collected without the addition of VTM	Yes
Teo *et al.* [43]	Feb-21	337	Confirmed COVID-19 patients and both symptomatic and asymptomatic unconfirmed patients	NPS	N1 and N2 genes ORF1ab gene	209/337, 62% 167/337, 49.6%	Not mentioned	Asked to tilt head back, clear both nose and throat and spit 2 ml into sterile container with 2 ml of RNA stabiliser fluid - asked to avoid eating/drinking/smoking at least 30 mins before collection	Not mentioned
Barat *et al.* [87]	Feb-21	459	Unconfirmed symptomatic patients	NPS or midturbinate swab	N gene (N1 and N2)	30/38, 81.5%	one saliva only, seven NPS/ midturbinate swab only	Asked to pool saliva in mouth for 30 s, and drool into sterile container until 3–5 ml was collected.	Yes
Rodríguez Flores *et al.* [61]	Mar-21	30	Confirmed COVID-19 patients	NPS	E and RdRP genes	26/30, 88.2%	four NPS only	Supervised oral saliva collection stored in a cooler after collection	Yes
Borghi *et al.* [73]	Mar-21	192 80 109	Adult cohort Asymptomatic cohort Child cohort	NPS	RdRP and N1 genes	60/86, 69.76% 9/12, 75% 26/27, 96.3%	one saliva only, 29 NPS only 3 NPS only six saliva only, one NPS only	A sterile dental cotton roll was used to collected saliva by holding in the mouth for 4 min total. Cotton roll was stored at room temperature until processing	No
Kernéis *et al.* [46]	Apr-21	372 1328 1383	Unconfirmed symptomatic and asymptomatic close contacts with three different saliva processing protocols MDI-1 MDI-2 Roche	NPS	N, S and ORF1ab N, S and ORF1ab E and ORF1ab	23% 94% 96%	66 saliva only, five NPS only	Not mentioned	No
De Santi *et al.* [47]	Oct-21	127 181	Hospitalised, symptomatic confirmed COVID-19 Unconfirmed asymptomatic	NPS	RdRP and N1 genes	82/87, 94.3%	nine saliva only (of which two were false positives), five NPS	Collect by passive drooling. Excluded if eating/drinking/oral hygiene/nasal sprays were consumed 30 min before collection	Mean difference not statistically significant
LeGoff *et al.* [48]	Oct-21	1718	Unconfirmed patients, one third of which were symptomatic	NPS	ORF1ab, N and S genes	93%	Not mentioned; although saliva detected more positives, 153, compared to only 110 for NPS, indicating 43 were saliva only	Self-collected saliva, patients were asked to swish around saliva in their mouths for 30 s before spitting - asked to avoid eating/drinking/smoking at least 30 mins before collection	Not mentioned

Some studies collected saliva in the early morning [19, 35, 38, 39, 55, 56] when viral load is believed to be highest and/or asked patients to refrain from eating/drinking/smoking and/or oral hygiene from 0.5 h to overnight before specimen collection [19, 35, 38, 39, 43, 45, 47, 48, 54–56, 60, 62, 65, 70, 71, 76] as this has been demonstrated to increase SARS-CoV-2 concentration in saliva [77–79] and for example mouth wash has been shown to reduce the SARS-CoV-2 viral load in saliva specimens [80, 81]. Whilst one study noted that they did not observe a difference in saliva specimen sensitivity when patients had eaten/drank/smoked/used oral hygiene within 30 min of sample collection [46], the general consensus in the majority of studies is that patients should avoid eating/drinking/smoking and/or oral hygiene before providing a saliva sample. This adds an extra layer of variability in saliva sample sensitivity compared to NPS, as such recommendations are not required nor have been shown to influence NPS specimen sensitivity (Fig. 1, Table 2).

**Table 2.** Strengths and weakness of NPS and saliva specimens

Specimen	Strengths	Limitations
NPS	Already established as the specimen of choice for the diagnosis of many viral respiratory infections [4, 5] Likely to be less variation in the sensitivity of the specimen if food/drink etc. is consumed before sample is taken Antigen tests are established for using NPS as their input	Uncomfortable and invasive specimen to collect Not suitable for children Requires trained HCWs in order for the sample to be collected Puts HCWs at risk of nosocomial infection Incorrect swabbing due to poor technique from administrator or resistance from the patient can impact sensitivity Not suitable for mass-testing/surveillance due to logistics and repeated sampling can cause adverse effects to the patient [13]
Saliva	Less invasive specimen to collect and therefore patients are more likely to get tested and give repeated samples Can be self-administered from home, so no trained HCWs or test sites are required Suitable for COVID-19 mass testing/ surveillance	No established collection, transport and processing protocol Time of day of sampling as well as food/drink/oral hygiene/smoking can impact the specimen’s sensitivity Antigen tests are not established for using saliva as their input

The transportation and storage of the saliva before processing also varied, with some adding VTM [15, 19, 35, 40, 56], UTM (universal transport medium) [59], amies medium [52], phosphate buffer saline (PBS) [55, 82] and/or storing at 4 °C/freezing [41, 45, 54, 55, 83–85] until processing and others using crude saliva and/or stored at room temperature [38, 39, 63, 86]. The studies that added VTM, UTM, amies medium or PBS to saliva samples had sensitivities that ranged from 77 % [55] to 91.7 % [15]. The studies that used VTM [15, 35, 40, 56], which is the transport medium used for NPS specimens, tended to have higher sensitivities than those that used other transport media [52, 55, 59, 82], although this may be due to other parameters of these studies such as that they [15, 35, 40, 56] all used POPS, which as aforementioned may potentially have a higher viral load compared to oral saliva, which was used for the other studies. Some studies have noted that crude saliva can be difficult and time-consuming to process due to its viscosity, sometimes congealing after collection and thus recommend the addition of dilutants such as VTM at collection or proteases (e.g. mucolyse or proteinase K) before processing to ensure saliva is amenable for pipetting and further downstream processing [59, 70, 76, 86, 87]. Although it is not noted in the studies that used POPS examined for this review, this may be a particular issue with this specimen type over oral saliva as the potential mixing with sputum during throat clearing to obtain the specimen could increase its viscosity. This is especially an issue when trying to automate the diagnostic protocol to increase testing speed and capacity, where one study that developed a robotic RNA extraction step noted significant issues in using saliva specimens for this due to the inherent variable nature and viscosity of saliva specimens [76]. In many studies, and indeed in most saliva diagnostic tests that have been granted EUAs by the US FDA, stabilisers and preservatives are added to saliva specimens in order to minimise RNA degradation and thus maximise SARS-CoV-2 detection [16]. However, others have concerns that the addition of VTM/other transport media and stabilisers/preservatives may dilute the sample and/or reduce its sensitivity [25, 54, 86]. A recent study has found that SARS-CoV-2 RNA remains stable and detectable without a statistically significant raise in Ct values in saliva specimens that have both not had preservatives/stabilisers added nor have been stored at refrigeration/freezing temperatures, negating the need for these processes in the diagnostic protocol, particularly if additives may interfere with/reduce diagnostic sensitivity [16]. This further highlights the applicability of saliva specimens for diagnosis particularly in resource-limiting regions where preservatives/stabilisers, as well as VTM which is required for NPS specimen collection, and/or refrigeration/freezing facilities may be limited.

Of the studies that mentioned how they stored their saliva samples, those that stored theirs at 4 °C [41, 45, 54, 83] had sensitivities ranging from 78.6 % [45] to 92 % [41], those that mentioned storing theirs at −80 °C [55, 84, 85] had sensitivities ranging from 77 % [55] to 94.5 % [84] and those that mentioned storing theirs at room temperature [38, 39, 63, 73, 86] had sensitivities ranging from 69.76 % [73] to 94.12 % [63]. Whilst freeze-thawing can reduce the concentration/integrity of RNA in samples [85, 88], a high sensitivity of 94.5 % of −80 °C stored saliva samples were obtained in Vaz *et al.* [84]. Additionally, Ott *et al.* did not notice a statistically significant rise in Ct values of saliva samples that were stored at room temperature over a number of days [16] and thus it remains unclear what is the optimum storage conditions of saliva as clearly high sensitivities can be achieved with storage at 4 °C, freezing (−20 °C or −80 °C) and room temperature. The US Centres for Disease Control and Prevention (CDC) guidelines advice storing NPS specimens at 2–8 °C for up 72 h before processing and if processing is delayed beyond that, to store the samples at −70 °C or below [89].

Thus, there is clearly great diversity in the saliva collection, transport and storage protocols being employed which could explain the discrepancies in saliva sensitivity between studies. Comparatively, there are fewer potential variables in collecting NPS due to a standard protocol being in place where all NPS are placed only in VTM after collection (Fig. 1), typically refrigerated before processing and patients consuming food/beverages etc. does not appear to have any influence on sensitivity. However, reduced sensitivity can be seen in NPS specimens if there is poor technique used by the administrator (i.e. if the swab does not reach all the way to the nasopharynx or has limited contact time at the nasopharynx thus reducing the chances of collecting the virus) [90], which may occur for example if the swab is self-administered such as in [91, 92], or if there is patient resistance due to uncomfortable and invasive nature of NPS collection (Fig. 1, Table 2). Czumbel *et al.* remark that in order for saliva data to be reliable and comparable, there must be standardisation of an optimal collection and processing protocol [25]. Otherwise, as Hung and colleagues observe, discrepancies between results may be as a result of poor specimen collection technique, such as in one study where saliva samples were taken directly from the opening of the salivary glands and thus only correctly identified four out of 31 confirmed COVID-19 cases [57, 79].

### Sensitivity of saliva versus nasopharyngeal swabs

Specimen sensitivity for COVID-19 diagnosis, especially in asymptomatic/mild cases, is vital for disease control as the consequences of false negative results can lead to otherwise preventable outbreaks [93]. Importantly, no diagnostic test has both 100 % sensitivity and specificity, with NPS only having a detection rate of around 63 % [94, 95], with an estimated false negative rate ranging from 20–38 % after symptom onset, which are increasingly more likely in the days before symptoms begin and during the convalescent phase [96]. As aforementioned, there is great heterogeneity in the saliva collection, transport and storage protocols used in studies examining saliva sensitivity. There is no difference in the processing protocols used for saliva specimens which can influence sensitivity of the assay. This is demonstrated by Kernéis *et al.,* where they experimented with three different protocols for saliva processing and saw drastic changes in sensitivity [46]. The MGI-1 protocol, which was designed for NPS specimens, only had a sensitivity of 23 % with saliva, however, when it was adjusted by changing the lysis buffer to better suit saliva specimens (MGI-2 protocol), the sensitivity increased to 94%, dramatically highlighting the importance and standardisation of an accurate protocol and how different protocols can influence saliva sensitivity.

In comparing saliva to NPS, most studies reported discordance where neither specimen detected all positive cases with saliva samples detecting SARS-CoV-2 RNA where NPS samples were negative and vice versa (Table 1). Jamal *et al.* note that in studies that involving confirmed COVID-19 patients diagnosed using NPS, there may be a bias towards subsequent NPS over other specimens being positive [55]. Furthermore, Czumbel *et al.* point out that this discordance may not be because one specimen is ‘better’ than the other, but could be as a result of the varying sites of SARS-CoV-2 replication during the course of infection, where it is thought to begin in the nasal mucosa, or potentially in the oral mucosa [34], and subsequently disseminate down the respiratory tract, therefore specimen sensitivity may depend on the spatiality and temporality of SARS-CoV-2 replication during the course of infection [25, 97]. Borghi *et al.* found that SARS-CoV-2 was detectable in two saliva samples 48 h before it was detected in an NPS from the same individual indicating viral replication may begin in the oropharynx [73]. A similar result was seen in Savela *et al.,* who found that although NPS had the higher viral loads overall, saliva was able to detect SARS-CoV-2 up to 4.5 days before NPS [92]. However, a limitation of this study was the small sample size (seven in total) and that the NPS were self-administered which could diminish sensitivity thus further work with a larger sample size is needed to confirm this. Such results also may explain the variation in viral loads between the two specimens, where some studies reported that saliva specimens had a higher cycle threshold (Ct) value signifying a lower viral load compared to NPS specimens whereas others reported the opposite (Table 1). De Santi *et al.* found that in a hospitalised symptomatic patient cohort, NPS and saliva specimen sensitivity decreased at a consistently similar rate as time progressed over a course of greater than 7 days from the patients' initial positive diagnostic result [47]. Whereas Wyllie *et al.* found that more saliva samples remained positive 10 days after initial diagnosis than their corresponding NPS samples [91]. Nonetheless, from these studies it would appear that the sensitivity of saliva and NPS decrease at least at a similar rate. To *et al.* found viral load to be highest in saliva earlier in infection and found it declined over the course of disease [15] similar to Zhu *et al.* who found viral load in saliva to peak 1 week after symptom onset followed by a steady decline, but in 8/20 patients it remained detectable greater than 14 days from symptom onset which interestingly, was not correlated with disease severity [98]. Another study found the positive percentage agreement (PPA) was highest between saliva and NPS when saliva was collected within 9 days of symptom presentation, 65.6–93.5 %, compared to saliva collected 10 days after symptom onset, 22.2–66.7 %, and saliva obtained from asymptomatic patients, 40–66.7 % [82]. Similarly Wong *et al.* reported a PPA of 96.6 % when saliva was collected within 7 days of symptom onset and discordance was more likely to occur when saliva was collected after 7 days [19].

It has been noted that positive NPS samples with a corresponding negative saliva sample had high Ct values thus indicating that the discordant saliva sample may be as a result of low viral loads which are below the detection limit of RT-qPCR [65, 86]. For example, De Santi *et al.* recorded nine saliva only positives and five NPS only positives [47]. This study used the RNA extraction-free protocol for saliva processing, SalivaDirect, developed by Vogels *et al.* [24] but were unique among the studies we looked at in that they verified their discordant results again, this time using the TaqPath COVID-19 CE-IVD RT-PCR kit and found that 7/9 of the discordant saliva results were indeed true positives that had not been detected in the corresponding NPS samples, with only two being false positives but these had had Ct values >35 in the SalivaDirect protocol. The corresponding five false negative saliva samples remained undetected were likely due to low viral loads in saliva as the corresponding NPS samples had Ct values >30.

In studies that compared saliva sensitivity to NPS in mild/asymptomatic patients, two studies in particular found lower saliva sensitivities at 53 % [49] and 68.6 % [50]. This lower sensitivity may be as a result of poor specimen collection as both studies poorly described saliva collection protocol. The former pooled saliva specimens and the latter used a novel saliva collection kit containing viricidal fluid and preservatives which may have affected sensitivity of detection. Another study with a diverse sample cohort of both symptomatic and asymptomatic individuals found that saliva also had much lower sensitivity compared to NPS at 51.9 % [60]. However, when this study only included saliva positive results with a Ct value ≤30, the sensitivity of saliva specimens increased to 91.6 % indicating low viral load may be a possible reason for this discordance between saliva and NPS specimens [60]. Conversely, other studies found much higher saliva sensitivity at 93 % [39], 92 % [41], and 91.37 % [99] in mild/asymptomatic patients. Wyllie *et al.* screened 495 asymptomatic HCWs and found 13 positive saliva samples of which only two had a corresponding positive NPS indicating saliva is more sensitive for detecting asymptomatic infection [91]. However, a limitation of this study is that the NPS were self-administered and thus may be more likely to be inaccurate due to the technical challenge and discomfort of a self-administered NPS.

Thus, it is difficult to directly compare saliva and NPS sensitivities between current studies due to their heterogeneity but from the research so far it would appear that saliva sensitivity is at least comparable to NPS, especially if saliva specimen collection is supervised [100]. Further research to conclude what conditions i.e. collection and processing protocol, time from symptom onset etc., optimise saliva specimen sensitivity is still required to fully determine the optimal saliva collection and processing protocol as it is clear that under certain conditions saliva is less reliable and in others it is equal to/more reliable than NPS.

### Saliva as a diagnostic specimen for mass-testing and COVID-19 surveillance

Mass testing has been advocated as a key for controlling the pandemic. As the easing of restrictions corresponds with a rise in cases, mass testing and surveillance is crucial for breaking this cycle as well as providing countries with opportunities to catch new variants before they become widespread. With NPS currently being unable to keep up with testing demands, saliva offers a number of advantages to facilitating mass testing. Firstly, it is cost-effective, with one study estimating processing 100 saliva specimens costs $8.24 compared to $104.87 for 100 NPS [19]. Secondly, it alleviates specimen collection ‘bottlenecks’ due to supply shortages and wait-times as it has the potential to be self-administered from home. Thirdly, the non-invasive nature of saliva collection means individuals are more likely to give repeated samples for surveillance and monitoring. Additionally, multiple saliva samples, for example from the same household, could be pooled together which could further relieve strains on testing facilities allowing 5–10 samples to be processed as one [76, 87, 101, 102]. Although, pooling of saliva samples was shown in these studies to slightly reduce detection sensitivity. Ultimately, whilst saliva specimens can relieve some of the pressures seen with NPS, the diagnosis step, RT-qPCR, still takes the same amount of time, labour and resources [49]. Whilst in theory, an RT-qPCR result should be available in a number of hours from sample collection, in reality, due to testing demand, resource availability etc. the lag time between testing and receiving diagnostic results can be up to 3 days with RT-qPCR-based tests [103] which may contribute to viral spread particularly if individuals are asymptomatic or are not self-isolating.

This has led to the development of a number of point-of-care tests (POCT) using saliva as the diagnostic specimen, such as reverse-transcription loop-mediated isothermal amplification (RT-LAMP) which unlike RT-qPCR does not require thermal cycling for nucleic acid amplification, and often excludes an RNA extraction step or uses a simpler RNA extraction method such as treatment with a protease followed by heat [104], allowing for rapid diagnosis in hospitals/clinical settings as well as in resource-limited settings as the only equipment required is a heat-block [104–107]. However, Taki *et al.* found that without an RNA extraction step, saliva RT-LAMP only had a sensitivity of 47 % but with an RNA extraction step it had a sensitivity of 100 % indicating this step is vital for RT-LAMP sensitivity [108]. Other POCTs, such as Cepheid’s Xpert Xpress SARS-CoV-2 assay, which has been granted an EUA by the US FDA [109], allows for rapid results within 35–45 min without any extensive sample processing steps and has been used with saliva specimens for SARS-CoV-2 diagnosis in some studies with very high sensitivity rates when compared to NPS specimens with RT-qPCR at 100 % [110], 95.92 % [64] and 89.7 % [56]. However, as Landry *et al.* note, there are limitations in the applicability of the Xpert Xpress SARS-CoV-2 assay to mass-testing due to the limited availability of GeneXpert systems and its unsuitability to process large numbers of samples [70].

The development of point-of-need tests (PONT) such as lateral flow, or rapid antigen, assays that detect SARS-CoV-2 antigens like spike protein [111] or nucleocapsid protein [112] have also been developed which allow for rapid, onsite diagnosis within 15–30 min without any laboratory equipment and thus could be performed in schools, businesses, concerts etc. [105]. The majority of these rapid antigen detection (RAD) tests have been designed with NPS as their input which of course carries the same disadvantages as with it being the specimen for RT-qPCR. Therefore, a number of studies have examined various RAD tests with saliva specimens as their input [82, 112–115]. However, all have found saliva in conjunction with the RAD test to be less sensitive than both NPS as the input for the RAD test and RT-qPCR. It is important to consider that these RAD tests are often validated and designed for the use of NPS and therefore might by biassed towards NPS over saliva or there may be inhibitory factors present in saliva which may explain the reduced sensitivity seen when saliva is the input specimen. Additionally, there is a general consensus that RAD tests are typically less sensitive than RT-qPCR, even when NPS is used as the input specimen [116, 117]. However, this should not be the primary focus of RAD tests as the purpose of their design is to have a rapid and accessible means of testing that would allow for the detection of otherwise undetected cases in social settings that could result in a preventable outbreak. Therefore, it is argued that the frequency that these PONTs are employed combined with their rapidity is more important than their sensitivity for COVID-19 surveillance [118]. That being said, a recent evaluation of a number of RAD tests by Public Health England found that the promising Innova lateral flow test, which uses a nasal swab as the specimen, successfully detected 79.2 % of COVID-19 cases when carried out by trained laboratory scientists but only successfully detected 58 % of COVID-19 cases when carried out by self-trained lay people which raises concerns that if the specificity of these tests is dependent on the level of training/expertise of the individual conducting the test it could result in false negative results when these tests are widely implemented to the public outside of clinical trials [119]. Whilst both POCT and PONT with saliva as the diagnostic specimen would allow for diagnosis in resource-limited regions/countries as well as holding promise for a return to normality allowing for rapid screening in social settings, further research and optimisation of these tests is still required to evaluate and optimise their sensitivity in order to maximise their COVID-19 mass-screening potential [82].

It is also important to mention that in addition to saliva being an alternative diagnostic specimen to NPS, it has been demonstrated to substitute as a much less invasive and more easily obtainable specimen to serum samples to measure seropositivity following COVID-19 infection, with comparable levels of antibodies, in particular IgG, between paired saliva and serum samples [120, 121]. Thus, the easily obtainable saliva specimen could potentially be applied for mass ‘sero’-surveillance in addition to mass-testing to measure the levels of active infection and immunity, both natural and vaccine acquired, in the community [121, 122]. As the end of the COVID-19 pandemic is far from being insight, an easily testable sample to measure immunity might be of particular importance in the future as the longevity of vaccine-acquired immunity to COVID-19 infection is unknown.

### Limitations

The use of saliva as a specimen for COVID-19 diagnosis is not without its limitations and as aforementioned, a major concern is its sensitivity which has been discussed above. However, other limitations of using saliva as a specimen include that several studies reported that a number of saliva specimens were of insufficient volume/quality for processing [41, 42, 53, 67, 76, 85, 86, 123]. Thus, as Leung *et al.* note, step-by-step instructions are vital for collection of saliva samples of sufficient quality and quantity [40]. However, Fernández-González *et al.* compared self-collected saliva with only written instructions provided verses trained HCW-supervised collection of saliva specimens and found that when supervised, saliva specimens had 86 % sensitivity compared to NPS whereas self-collected specimens only had 66.7 % sensitivity, indicating that perhaps supervised collection is necessary to maximise saliva specimen quality which may limit the implementation of ‘from home’ self-collected saliva samples [100]. Interestingly, as Sagredo-Olivares *et al.* note, some studies reported employing telemedicine sessions with trained HCWs to ensure saliva specimen collection is done correctly and sufficiently from home [45, 124–126], which may offer a number of advantages to limit nosocomial infections and testing wait-time bottlenecks allowing at-home specimen collection whilst ensuring saliva specimen integrity [127]. Two studies also noted that whilst saliva sample collection has the potential to be self-administered by the patient in the absence of a trained HCW, it is important that the tube is decontaminated after as it has been noted that patients can spill the sample onto the outside of the tube, which if it contains infectious particles could put individuals processing the sample at risk of infection [47, 61]. Finally, saliva is not a suitable specimen for intubated patients as well as for those who may have difficulty producing saliva due to co-morbidities and/or medications [49, 54, 59].

As previously outlined, the variation in specimen collection and processing protocols between studies, with variations even within some studies [58], is a major limitation of the literature thus far and possibly explains the discordance in results seen between studies. It’s difficult to compare the sensitivities/accuracy between studies if the experimental parameters such as specimen collection protocol, transport and storage, PCR amplification targets, patients’ status etc. are not the same. In the majority, study design was also poor with small sample sizes, lack of blinding and of control-group samples with many studies only containing confirmed COVID-19 patients [128]. In order for fair comparisons to be made, there needs to be diversity in the sample cohort, particularly containing mild/asymptomatic patients who make up 80 % of COVID-19 cases and are likely to have a lower viral load [129, 130]. The implementation of a standard processing protocol, such as the SalivaDirect protocol, which accommodates the use of different kits/reagents and eliminates the RNA extraction step, could reduce the variation in sensitivities that occur due to differences in sample processing [24]. This protocol has been verified by other research groups, where Rodríguez Flores *et al.* found the SalivaDirect protocol to have 88 % sensitivity compared to NPS [61] and De Santi *et al.* found it to have 94.3 % sensitivity compared with NPS [47]. Interestingly, the former study also used the SalivaDirect protocol with NPS and found this to be slightly more sensitive, with 93.7 % sensitivity, than when saliva was used as the input for the SalivaDirect protocol which would indicate that NPS is the slightly more sensitive specimen due to higher viral load in this sample type, indicated by lower Ct values [61].

Also, a number of studies are not peer-reviewed [24, 38, 46, 49, 54, 58, 65, 76, 92, 99] and therefore these results should be interpreted cautiously and more rigorously conducted studies should be carried out before definitive conclusions can be made [10, 79].

### Conclusion

It is going to be a significant amount of time before enough of the world’s population are vaccinated and a sustained reduction of COVID-19 incidence will be seen. Therefore, identification of positive cases and the subsequent isolation of these individuals remains to be the most effective control mechanism of preventing COVID-19 spread thus far. Thus, a test that is accurate, convenient, cost-effective, is not dependent on specific supplies such as swabs, has patient acceptability and is suitable for mass-testing is required. NPS have failed to satisfy these requirements and saliva has emerged as a promising replacement and/or alternative specimen with a number of advantages. Whilst concerns over sensitivity of saliva specimens compared to NPS have been raised, it is clear that under the right collection, transport/storage and processing conditions saliva can be as sensitive and in some studies more sensitive than NPS specimens for COVID-19 diagnosis. Further research into the development of an optimum collection and processing protocol could attenuate the variation in sensitivities seen between studies. Nonetheless, a potential slight reduction in sensitivity compared to the reference standard might still be acceptable in specific testing environments such as mass-testing/surveillance and resource-limited settings, especially when considering the numerous advantages of saliva specimens [10].
